# The Transition to an Entirely Digital Immunization Registry in Ha Noi Province and Son La Province, Vietnam: Readiness Assessment Study

**DOI:** 10.2196/28096

**Published:** 2021-10-25

**Authors:** Hong Duong, Sang Dao, Huyen Dang, Linh Nguyen, Tuan Ngo, Trung Nguyen, Lan Anh Tran, Doan Nguyen, Maya Rivera, Nga Nguyen

**Affiliations:** 1 National Institute of Hygiene and Epidemiology Hanoi Vietnam; 2 PATH Vietnam Hanoi Vietnam

**Keywords:** immunizations, immunization registry, readiness assessment, electronic immunization records, Vietnam

## Abstract

**Background:**

Vietnam is one of the first low- to middle-income countries to develop and implement a national-scale electronic immunization registry. This system was finalized into the National Immunization Information System (NIIS) and scaled up to a national-level system in 2017. As a result, immunization coverage and the timeliness of vaccinations have drastically improved. The time spent on planning and reporting vaccinations has drastically reduced; as a result, vaccination planning and reporting has become more accurate and effective. However, to date, end users have been tasked with managing both the NIIS and paper-based systems in parallel until a formal assessment of the readiness to fully transition to the NIIS is conducted.

**Objective:**

This study aims to evaluate the readiness to move to an entirely digital NIIS in 2 provinces of Vietnam—Ha Noi and Son La.

**Methods:**

All health facilities were surveyed to assess their infrastructure, capacity, and need for human resources. NIIS end users were observed and interviewed to evaluate their NIIS knowledge and skill sets. Data from immunization cards and facility paper-based logbooks were compared with data from the NIIS, and vaccine stocks at selected facilities were tallied and compared with data from the NIIS.

**Results:**

Of the 990 health facilities evaluated, most used the NIIS to enter and track immunizations (987/990, 99.7%) and vaccine stocks (889/990, 90.8%). Most had stable electricity (971/990, 98.1%), at least 1 computer (986/990, 99.6%), and ≥2 trained NIIS end users (825/990, 83.3%). End users reported that the NIIS supported them in managing and reporting immunization data and saving them time (725/767, 94.5%). Although many end users were able to perform basic skills, almost half struggled with performing more complex tasks. Immunization data were compiled from the NIIS and immunization cards (338/378, 89.4%) and paper-based logbooks (254/269, 94.4%). However, only 54.5% (206/378) of immunization IDs matched, 57% (13/23) of Bacillus Calmette-Guérin vaccination records were accurate, and 70% (21/30) of the facilities had consistent physical vaccine stock balances. The feedback received from NIIS end users suggests that more supportive supervision, frequent refresher training for strengthening their skill sets, and detailed standardized guides for improving data quality are needed.

**Conclusions:**

The readiness to transition to a digital system is promising; however, additional resources are required to address the timeliness, completeness, and accuracy of the data.

## Introduction

### Background

Electronic immunization registries (EIRs) enable the collection and consolidation of immunization coverage and can therefore be an effective tool in the planning and delivery of immunization services [1,2]. EIRs are becoming more widespread in low- to middle-income countries because of more accessible and cheaper computer technologies [2]. Several studies have shown that the implementation of such systems improves data accuracy and timeliness and assists in identifying areas of low vaccine coverage and children who have missed vaccinations and as a result, saves health care workers a substantial amount of time [3,4]. However, the success and adoption of such systems are also contingent upon their ability to widely implement and successfully adopt these tools at a national scale [5-7]. The success of scale up of these systems is also highly contingent upon several factors such as adequate equipment, infrastructure, human resources and skills, reliable power supply, funding, and ongoing process evaluations [8].

Nonetheless, Vietnam is one of the first low- to middle-income countries to develop and implement a national-scale EIR. Beginning in 2012, PATH and the Vietnam National Expanded Program on Immunization (EPI) developed and piloted a digital immunization registry, ImmReg, in 1 district of the Ben Tre province. Between 2014 and 2015, ImmReg was integrated with VaxTrak (a vaccine tracking software) into a comprehensive software for the National EPI and implemented in the entire Ben Tre province. During 2016 and 2018, leveraging government-led efforts and private sector partnerships, this system was finalized into the National Immunization Information System (NIIS) and scaled up to a national-level system. Since its launch, the NIIS has been used in 99.8% of facilities across provinces and their districts nationwide, and >20 million children and women have been registered in the system. As a result, immunization coverage and timeliness of vaccinations have drastically improved [4]. Using the NIIS has also alleviated the burden on immunization officers across all levels. The time spent on planning and reporting vaccinations has drastically reduced, and as a result, it is more accurate and effective. A timely, complete, and transparent database has provided higher-level officers (district, provincial, and national) comprehensive information to better guide and strategically plan to improve immunization coverage, as well as respond promptly in cases of emergency.

However, to date, end users have been tasked with managing both NIIS and paper-based systems in parallel until a formal assessment on the readiness to fully transition to the NIIS is conducted. Even though this parallel process was burdensome for end users, it was an important and intentional part of the rollout process to troubleshoot any unanticipated issues with the NIIS and allowed end users to transition to the new digital registries.

### Goal and Objectives

Thus, the main goal of this study is to evaluate and compare the readiness of 2 provinces in Vietnam in moving entirely from a paper-based to paperless system for immunization records, with an emphasis on accuracy, completeness, consistency, timeliness, and data quality.

The following objectives are used to determine readiness to transition to the NIIS: (1) assess the infrastructure, capacity, need for human resources, and indicators for data accuracy; (2) assess NIIS end users’ perceptions and feedback; (3) evaluate the data quality of the NIIS; (4) compare the accuracy of stock data that were physically counted to stock data in the NIIS; and (5) evaluate the knowledge and skills of NIIS end users.

## Methods

### Study Area

This study was conducted in 2 provinces in Vietnam, Ha Noi City and Son La. These provinces were selected as they represented varying levels of readiness with respect to geography (urban, semiurban, rural, and mountainous areas), population density, facility type (fee-based, private, and public), and facility level (national, provincial, district, and commune). Ha Noi is the capital city and mostly urban with a large and growing population, high immigration rate, a good infrastructure system, and a higher number of private sector and fee-based facilities. In comparison, Son La is a primarily bordered mountainous province with limited resources and fee-based facilities.

### Study Design and Data Collection

Table 1 shows the research methods used to address each specific objective as described below, including the sampling strategy, data collection method, and type of data collected. All data were collected via the Kobo toolbox software [9], which identified data errors and missing data via built-in programmed checks.

**Table 1:** Sampling strategy, data collection methods, and types of data collected for addressing the five study objectives.

Objective	Sampling strategy	Data collection methods	Type of data collected
1. Assess and compare infrastructure, capacity, and human resources needs in 2 provinces that represent different levels of readiness	All immunization health facilities (N=1026) in Ha Noi and Son La Provinces	Self-filled forms in Kobo toolbox sent via email	Immunization services provided: EPI^a^, non-EPI, and hepatitis B birth dose vaccine coverage Infrastructure conditions: electricity, computers, type of internet connection, barcode printers, and readers Current NIIS^b^ use: NIIS log-in and username End users implementing the NIIS: number of people trained in use of NIIS or need for additional trainings Indicators for data accuracy: data entry for immunizations and stock balances
2. Assess NIIS end users’ perceptions and feedback	A total of 767 districts and communes were purposively selected	Self-filled forms in Kobo toolbox sent via email	Time spent planning and reporting before and after NIIS Perceptions of NIIS system on managing data and quality of data Feedback for system improvement
3A. Evaluate data quality between immunization cards and the NIIS	Son La: All children born after July 1, 2017, purposefully selected from 1 village in each of the 6 selected communes^c^ Ha Noi: 7^d^ children born after July 1, 2017, in each village or living quarter identified door-to-door, in each of the 12 selected communes^c^	Review and compare data between immunization cards and the NIIS	Immunization data on immunization cards of selected children Immunization data in the NIIS of selected children
3B. Evaluate data quality between paper-based records and the NIIS	20 randomly selected children from each of 14^e^ purposefully selected fee-based facilities and hospitals	Review and compare data between paper-based logbooks at health facilities and the NIIS	Immunization data written in the facility paper-based logbook of selected children Immunization data in the NIIS of selected children
3C. Evaluate data quality between paper-based records and the NIIS for BCG^f^ vaccination administered in previous month	18 randomly selected commune health centers and district health centers	Review and compare count of BCG immunizations among children in facility paper-based logbooks and in NIIS for previous month	Number of children vaccinated in previous month with the BCG vaccine per paper-based logbook Number of children vaccinated in previous month with the BCG vaccine in the NIIS
4. Compare the accuracy of stock data that were physically counted to stock data in the NIIS	Random selection of 3 vaccine lots in 30 health facilities with established NIIS system	Review of actual stock of 3 randomly selected vaccine lots and in NIIS	Physical stock balance of selected lots Stock balance per NIIS of selected lots
5. Evaluate the knowledge and skills of NIIS end users	Convenience sampling of 1 or 2 NIIS end users from each purposefully selected fee-based facility and hospital	Observation and interviews with NIIS end users	NIIS knowledge and skills of end users using the three modules: immunization registry, stock management, and reporting

^a^EPI: Expanded Program on Immunization.

^b^NIIS: National Immunization Information System.

^c^Overall, 6 out of 30 districts in Ha Noi and 3 out of 12 districts in Son La.

^d^Due to the large population size in Ha Noi province, the World Health Organization’s 30-clusters methodology was applied.

^e^One hospital that did not use the NIIS at the time of survey was excluded.

^f^BCG: Bacillus Calmette-Guérin.

### Objective 1

To describe and compare the characteristics of immunization facilities, human resources, and data accuracy indicators, all immunization health facilities in Ha Noi and Son La provinces were emailed a web-based survey about the immunization services provided; infrastructure conditions; current NIIS use; number of NIIS trained end users, including health care workers and managers at different levels, and at different types of facilities such as commune health centers, hospitals, and fee-based immunization facilities; implementing the NIIS; and NIIS acceptance and feedback. The number and frequency by province were evaluated and compared using the Wilcoxon Mann-Whitney test.

### Objective 2

To evaluate perceptions and feedback, the NIIS end users were purposively selected from districts and communes as follows: (1) of the 30 districts in Ha Noi, 6 (20%) were selected to represent a mix of population densities, facility types (fee-based, private, and public), and geographical areas (urban, semiurban, and rural); (2) of the 12 districts in Son La, 3 (25%) were selected to represent urban, rural, and mountainous areas; (3) 2 communes within each district, 1 to represent the smooth operation of the NIIS and the other to represent a commune with challenges were selected; (4) 6 hospitals in Ha Noi to represent different levels and types (urban, rural, private, and public) were selected; if there was >1 hospital in each category, then the hospital with the highest number of newborns in 2018 served as a substitute for purposively selecting high-volume hospitals; in Son La, 3 hospitals representative of urban and rural hospitals were selected; and (5) 2 fee-based hospitals and 1 public facility were selected in Ha Noi, and 3 fee-based facilities were selected in Son La. The NIIS end users were asked a series of survey questions to estimate the time it took them to plan and report on immunization before and after NIIS implementation and to evaluate their acceptability of the NIIS and its data quality that were compiled and analyzed. In addition, users’ feedback on how the NIIS system could be improved was elicited via open-ended questions; common themes were grouped and frequencies were noted to help the Ministry of Health (MOH) and software developers improve the NIIS.

### Objective 3

To evaluate data quality between immunization cards and NIIS in objective 3A, all children born between July 1, 2017, and the date of data collection were purposefully selected from 1 village in each of the 6 representative selected communes in Son La. In Ha Noi, 7 children born between July 1, 2017, and the date of data collection were selected from each village or living quarter using the World Health Organization’s 30-clusters methodology [10] in each of the 12 representative selected communes.

To evaluate the data quality between paper-based records and NIIS in objective 3B, 20 children from each of 14 purposefully selected fee-based facilities and hospitals in both provinces were randomly selected, and their records were compared. In addition, in purposively selected commune health centers and district health centers representative of facilities with and without NIIS implementation challenges, the number of children who received a Bacillus Calmette-Guérin (BCG) vaccination in the previous month per paper-based logbook at facilities was compared with the NIIS.

### Objective 4

To evaluate vaccine stock accuracy, stock balances of 3 vaccine lots were randomly selected from 30 health facilities with available vaccine stocks across both provinces that had an established NIIS, and physical stock and NIIS counts were compared.

### Objective 5

To address the knowledge and skills of NIIS end users, 1 or 2 NIIS end users from each purposefully selected fee-based facility and hospital were observed and interviewed on three modules: immunization registry, stock management, and reporting. Observations were used to evaluate end user skills, and interviews were used to evaluate end user knowledge.

### Statistical Analyses

Descriptive analyses, including counts, proportions, and means, were estimated for each objective and stratified by province where applicable. Responses from open-ended questions were counted and tabulated. All statistical analyses were conducted in Stata 14 (StataCorp LLC).

### Ethics

The study procedures were reviewed and received a nonresearch determination by PATH because the activity does not meet the definition of *research*, as defined in 45 Code of Federal Regulation 46.102(l). Interviewers and supervisors were trained on interview techniques, data collection procedures and tools, ethical issues (such as how to protect the identities of study participants where applicable and how to secure all the data collected), and quality control. Interviewees were informed of the study’s objectives and their rights of participation. Verbal consent was obtained from all participants.

### Ethical Approval

This research protocol was reviewed and determined as not human subjects research by the PATH research determination committee. Before the interviews, all participants were informed and received an explanation of the scope and purpose of the study, the right to participate, and confidentiality of their personal information. No personal information was provided.

## Results

### Objective 1

There were a total of 1026 immunization health facilities in Ha Noi and Son La provinces; 96.49% (990/1026) of health facilities completed the initial survey about infrastructure, capacity, and the need for human resources. Of the 990 facilities, 747 (75.5%) were in Ha Noi and 243 (24.5%) were in Son La. Table 2 shows the characteristics of these facilities by province.

**Table 2:** Characteristics of immunization facilities that completed the web-based survey, human resources, and data accuracy indicators by province (N=990).

Type of facilities		Total	Ha Noi (n=747)	Son La (n=243)	*P* value^a^
Commune health center, n (%)		788 (79.6)	584 (78.2)	204 (83.9)	.04
District health center, n (%)		42 (4.2)	30 (4)	12 (4.9)	N/A^b^
Fee-based facilities, n (%)		72 (7.3)	59 (7.9)	13 (5.4)	N/A
Government hospitals (center, provincial, and district), n (%)		61 (6.1)	49 (6.5)	12 (4.9)	N/A
Private hospitals, n (%)		23 (2.3)	22 (2.9)	1 (0.4)	N/A
Other, n (%)		4 (0.4)	3 (0.4)	1 (0.4)	N/A
**Vaccination services provided, n (%)**					
	EPI^c,d^ vaccines only	548 (55.4)	354 (47.4)	194 (79.8)	<.001
	Non-EPI^a^ vaccines only	92 (9.3)	79 (10.6)	13 (5.4)	N/A
	Both EPI and non-EPI vaccines	224 (22.7)	222 (29.8)	2 (0.8)	N/A
	Hepatitis B Birth dose	51 (5.2)	42 (5.6)	9 (3.7)	N/A
	EPI vaccines and Hepatitis B Birth dose	26 (2.6)	10 (1.3)	16 (6.6)	N/A
**Infrastructure**					
	Has stable electricity, n (%)	971 (98.1)	747 (100)	224 (92.2)	<.001
	Has at least one computer, n (%)	986 (99.6)	747 (100)	239 (98.4)	<.001
	Number of computers, mean (SD)^e^	2.9 (2.3)	3.0 (2.4)	2.5 (1.9)	<.001
	Number of computers ready for NIIS^f^, mean (SD)	1.9 (1.5)	2.0 (1.7)	1.7 (0.9)	.04
	Has cable or Wi-Fi internet connection, n (%)	944 (95.3)	747 (100)	197 (81.1)	<.001
	Has 3G or 3G internet connection, n (%)	46 (4.7)	0 (0)	47 (18.9)	N/A
	Has a barcode printer, n (%)	89 (8.9)	38 (5.1)	51 (20.9)	<.001
	Has a barcode reader, n (%)	85 (8.6)	74 (9.9)	11 (4.5)	.009
**Current NIIS use, n (%)**					
	Has NIIS log-in username	990 (100)	747 (100)	243 (100)	N/A
	Currently using the NIIS system	987 (99.7)	744 (99.6)	243 (100)	.32
	Provided personal immunization ID	881 (89.3)	740 (99.5)	141 (58)	<.001
	Have ≥2 end users use NIIS	766 (77.4)	610 (81.7)	156 (64.2)	<.001
**NIIS trained end users**					
	Has only 1 health worker trained on NIIS, n (%)	147 (14.9)	105 (14.1)	42 (17.3)	.22
	Have ≥2 end users trained on NIIS, n (%)	825 (83.3)	633 (84.7)	192 (79)	.04
	Additional training on the NIIS not required, n (%)	165 (16.7)	140 (18.7)	26 (10.6)	.002
	Number of end users trained on NIIS, mean (SD)	3.1 (2.4)	3.3 (2.7)	2.6 (1.5)	.004
	Number of end users that need additional training on NIIS, mean (SD)	3.7 (2.4)	3.7 (2.4)	3.7 (2.2)	.27
**Indicators of data accuracy^g^, n (%)**					
	Data entered during the immunization session	697 (70.4)	644 (86.2)	53 (21.8)	<.001
	Data entered after the immunization session	290 (29.3)	101 (13.5)	189 (77.7)	N/A
	Data are not entered	3 (0.3)	2 (0.3)	1 (0.4)	N/A
	Entered stock data into NIIS	899 (90.8)	677 (90.6)	222 (91.4)	.73
	NIIS stock balance is accurate	884 (89.3)	679 (90.9)	205 (84.4)	.62
	NIIS immunization data matched paper records	753 (76.1)	612 (81.9)	141 (58.0)	<.001
	Correctly reported number of fully immunized children	762 (76.9)	612(81.9)	150 (61.7)	<.001

^a^Non–Expanded Program on Immunization vaccines are not in the list of Expanded Program on Immunization vaccines, and clients have to pay from their own pocket for vaccination.

^b^N/A: not applicable; too few estimates to determine significance.

^c^Expanded Program on Immunization vaccines are vaccines introduced and provided to people (children and women) free of charge.

^d^EPI: Expanded Program on Immunization.

^e^Wilcoxon Mann-Whitney test was applied.

^f^NIIS: National Immunization Information System.

^g^Per the Ministry of Health 4-step immunization process (among those who are using the NIIS in the last month).

The distribution of facilities differed in both provinces (*P*=.04); although 79.6% (788/990) were commune health centers, 9.5% (71/747) of facilities in Ha Noi were government and private hospitals compared with 5.3% (13/243) of facilities in Son La. The type of vaccination services provided was significantly different between the provinces (*P*<.001). EPI vaccines were the most common type of service provided: 79.8% (194/243) of facilities in Son La compared with 47.4% (354/747) of facilities in Ha Noi. Among the 990 health facilities, 971 (98.1%) had stable electricity, and 986 (99.6%) had at least one computer (Table 2). Compared with Son La, facilities in Ha Noi had a higher mean number of computers (3.0, SD 2.4 vs 2.5, SD 1.9; *P*<.001), higher mean number of computers ready for NIIS (2.0, SD 1.7 vs 1.7, SD 0.9; *P*=.04), more cable or Wi-Fi internet connections (747/747, 100% vs 197/243, 81.1%; *P*<.001), and more barcode readers (74/747, 9.9% vs 11/243, 4.5%; *P*=.009) but less barcode printers (38/747, 5.1% vs 51/243, 21%; *P*<.001). In addition, all 990 facilities had a NIIS log-in username, and 99.7% (987/990) reported having used the NIIS. A total of 3 hospitals in Ha Noi did not use NIIS. Of the 990 facilities, 881 (89%) provided personal immunization IDs to newborn babies and clients; this was higher in Ha Noi than in Son La (740/747, 99.5% vs 141/243, 58%; *P*<.001).

Of the 990 facilities, 825 (83.3%) had >2 end users trained on NIIS and 165 (16.7%) did not require additional training (Table 2). However, there was a significant difference (*P*=.004) in the mean number of trained NIIS end users in Ha Noi as compared with Son La (3.3, SD 2.7 vs 2.6, SD 1.5), even though the mean number of end users requiring additional training was the same in both provinces (mean 3.7, SD 2.4 in Hanoi and mean 3.7, SD 2.2 in Son La).

Data entry differed by province (Table 2); 86.2% (644/747) of facilities in Ha Noi entered the data during the immunization session, compared with 21.8% (53/243) of facilities in Son La (*P*<.001). For data accuracy, of the total 990 facilities, 899 (90.8%) entered stock data into the NIIS, and 884 (89.3%) reported that their stock balance accurately reflected their actual physical stock. Although most facilities reported NIIS immunization data matched paper records and correctly reported the number of children fully immunized, Ha Noi had a significantly higher proportion than those in Son La (612/747, 81.9% vs 141/243, 58%; *P*<.001).

### Objective 2

A total of 767 NIIS end users, 580 (75.6%) from Ha Noi and 187 (24.4%) from Son La, provided estimates of monthly time spent planning and reporting on immunization before and after NIIS. Before NIIS was launched, it took end users an average of 10.9 hours to prepare immunization plans and 13.2 hours to report on the immunization data in their facility. After NIIS was launched, the average amount of time spent on planning and reporting was 3.1 hours and 4.5 hours, respectively. These time estimates were significantly different and resulted in 72% (7.8/10.9) of the time saved on planning and 66% (8.7/13.2) of the time saved on reporting. Similar results were found for each province (data not shown). In addition, among 767 end users, 725 (94.5%) reported that the NIIS supported them in managing and reporting the immunization data in their respective facilities; however, 9 (1.2%) reported that the system did not support them, and 33 (4.3%) reported that it increased their workload.

Feedback from end users for system improvement identified that there is still a need to develop detailed guidelines and standard operating procedures for all components of the NIIS, from registration to reporting; provide more direction and supervision to allow end users to strengthen their skill sets; offer annual refresher training when new functions are added to the system; upgrade software, technology, and internet connections; and improve functions to easily identify and resolve errors. End users also reported that simplifying the number of indicators tracked, reducing the reporting forms, and ultimately eliminating the need to maintain parallel systems would help improve data quality and encourage them to use the NIIS more frequently for planning and reporting.

### Objective 3

Table 3 shows the indicators used to assess the data quality between the immunization cards and NIIS. A total of 378 immunization cards of children were evaluated; of the 378 cards, 229 (60.6%) were from Ha Noi, and 149 (39.4%) were from Son La. Overall, 62.4% (236/378) of immunization cards had an attached or written NIIS ID. Of the 378 immunization cards, 338 (89.4%) were registered in the NIIS, 206 (54.5%) had NIIS IDs recorded on both their immunization cards and NIIS records, 330 (87.3%) had demographic information that matched with NIIS, 372 (98.4%) of immunizations received since birth were recorded on the immunization cards, and 360 (95.2%) were entered into the NIIS but only 335 (88.6%) of immunizations recorded on both immunization cards and the NIIS matched. These indicators were all statistically different based on the province.

**Table 3:** Data Quality Indicators used to compare National Immunization Information System (NIIS) and immunization cards (N=378).

Immunization cards		Total	Ha Noi (n=229)	Son La (n=149)	*P* value
**Indicators, n (%)**					
	Immunization card had an NIIS ID	236 (62.4)	202 (88.2)	34 (22.8)	<.001
	Child was registered in the NIIS	338 (89.4)	217 (94.8)	121 (81.2)	<.001
	Both immunization card and NIIS had an NIIS ID	206 (54.5)	179 (78.2)	27 (18.1)	<.001
	Demographic information on immunization cards and NIIS matched	330 (87.3)	209 (91.3)	121 (81.2)	.01
	Immunizations recorded on immunization card	372 (98.4)	226 (98.6)	146 (97.9)	.02
	Immunizations recorded in the NIIS	360 (95.2)	224 (97.8)	136 (91.3)	<.001
	Immunizations recorded on immunization cards and NIIS matched	335 (88.6)	217 (94.8)	117 (78.5)	<.001

The data quality indicators used to compare the facilities’ paper-based logbooks and NIIS records for 269 children are shown in Table 4. Of these 269 records, 254 (94.4%) were registered in the NIIS, 251 (93.3%) had immunization data entered into the NIIS, and 242 (89.9%) had matching immunization data between the paper-based logbook and the NIIS. The proportions of the immunization indicators in Ha Noi were significantly higher than those in Son La (*P*<.001 and *P*=.03). There was no difference in immunization registration. When comparing the number of BCG vaccinations recorded in the logbook and NIIS in the previous month, only 57% (13/23) of facilities had accurate data.

**Table 4:** Data quality indicators used to compare National Immunization Information System (NIIS) and facility paper-based logbooks (N=269).

Facility paper-based logbook			Total		Ha Noi (n=196)		Son La (n=73)		*P* value
**Indicators, n (%)**									
	Child was registered in the NIIS	254 (94.4)		186 (94.9)		68 (93)		.58	
	Immunizations recorded in the NIIS	251 (93.3)		188 (95.9)		63 (86)		<.001	
	Immunizations recorded on paper-based logbooks and NIIS system matched	242 (89.9)		181 (92.4)		61 (84)		.03	

### Objective 4

Among 30 health facilities, 19 (63%) in Ha Noi and 11 (37%) in Son La, 18 (60%) facilities in Ha Noi tracked vaccine and supplies of 3 randomly selected lots in NIIS, as compared with 8 (27%) facilities in Son La (Table 5). However, only 84% (16/19) and 45% (5/11) of facilities had physical stock balances that matched the NIIS in Ha Noi and Son La, respectively.

**Table 5:** Data quality indicators used to compare National Immunization Information System (NIIS) and physical stock balances by province (N=30).

Physical stock		Total	Ha Noi (n=19)	Son La (n=11)	*P* value
**Indicators, (%)**					
	Vaccine and supplies of 3 lots tracked in the NIIS	26 (87)	18 (95)	8 (72)	.13
	Physical vaccine stock balances of 3 lots matched NIIS	21 (70)	16 (84)	5 (46)	.04

### Objective 5

The knowledge and skills of end users on the NIIS immunization registry, stock management, and reporting modules are shown in Table 6. Overall, a direct relationship between end users’ knowledge and skills was observed. Most end users were able to perform basic skills such as registering a client, entering data following a 4-step procedure in the NIIS, and updating personal information for an existing client. However, almost half struggled with more complex tasks, such as creating a vaccination plan by type of vaccine, deactivating immunization reminders, and identifying and eliminating duplicates. Similar results were observed across both provinces.

**Table 6:** Overall knowledge and skills related to the National Immunization Information System (NIIS) among end users on the NIIS main modules.

Function		Knowledge, n (%)	Skills, n (%)	Correlation	*P* value
**Immunization registry**		51 (100)	51 (100)	N/A^a^	N/A
	Register clients	48 (94)	49 (96)	0.8	<.001
	Search a client	36 (71)	35 (69)	0.86	<.001
	Search a newborn registered by a hospital	24 (47)	23 (45)	0.94	<.001
	Update personal information for an existing client	40 (79)	40 (78)	1	<.001
	Enter data following 4-step procedure in the system	45 (88)	44 (86)	0.88	<.001
	Add a migrant child from a given location into the list of children due for vaccination^b^	22 (52)	23 (55)	0.95	<.001
	Deactivate immunization schedule reminder of a child^b^	24 (57)	22 (52)	0.90	<.001
	Create a vaccination plan for children by vaccines^b^	25 (60)	24 (57)	0.95	<.001
	Duplication filter^b^	24 (57)	23 (55)	0.86	<.001
	Duplication handling^b^	23 (55)	22 (52)	0.95	<.001
**Stock management**		51 (100)	51 (100)	N/A	N/A
	Create a stock receipt from a vaccine distributor	19 (37)	18 (35)	0.96	<.001
	Create a stock receipt from higher level^b^	23 (55)	23 (55)	1	<.001
	Create a dispatch for low level^c^	7 (78)	7 (78)	1	<.001
	Create a vaccine use voucher	30 (59)	31 (61)	0.96	<.001
**Report only applied for CDC^d^, DHC^e^, and CHCs^f^**		26 (100)	26 (100)	N/A	N/A
	Generate and export monthly immunization report for children aged under 1 year	19 (73)	18 (69)	0.91	<.001
	Generate and export monthly immunization report for children aged over 1 year	17 (65)	16 (62)	0.92	<.001

^a^N/A: not applicable.

^b^Applied for Center for Disease Control and Prevention, district health center, commune health center, and hospitals (overall: n=42; Ha Noi: n=32; Son La: n=10).

^c^Applied for Center for Disease Control and Prevention and district health center (overall: n=9; Ha Noi: n=6; Son La: n=3).

^d^CDC: Center for Disease Control and Prevention.

^e^DHC: district health center.

^f^CHC: commune health center.

## Discussion

### Principal Findings

The readiness to transition to an entirely digital immunization reporting system in 2 provinces, Ha Noi and Son La, in Vietnam, was promising. Overall, there was a high level of use of the NIIS for immunization registry (987/990, 99.7%) and for tracking vaccine stock data (889/990, 90.8%). A total of 3 hospitals in Ha Noi did not use NIIS because they did not provide any vaccinations to newborns, or they used their own system to record immunization data. Evidence suggests that ownership and acceptability are critical components that facilitate the use and scale up [11]. Given that the NIIS is an integrated system with established standards to support and guide transition and scale up, it is imperative that provincial health authorities encourage all facilities to use the NIIS, or at least share data with the NIIS, especially if they run a facility-owned information system.

Most facilities in both provinces had sufficient infrastructures, such as stable electricity, at least one computer, and cable or Wi-Fi internet connections. In Son La, fewer computers were ready for NIIS and 18.9% (47/243) of facilities were still using a 3G or 4G internet connection. In addition, there were few barcode readers and printers across all the facilities. This was primarily because of limited resources, where programs within the facility often must share the only computer available and the limited resources available to upgrade their internet connection. To encourage the uptake and sustainability of systems such as the NIIS, budgets should be revised so that related expenses, such as power supply, critical equipment, and internet connection needed to support NIIS, can be procured [12-14]. Moreover, planning and making provisions for possible offline data entry in the interim could be beneficial in areas with poor internet connections or unstable power supplies [13].

Sufficient training is essential for end users to adopt and use digital health solutions, such as the NIIS [15]. To ensure that NIIS is sustainable in that immunization data are entered and updated in a timely manner, each facility should have at least two trained end users to use NIIS. However, only 77.4% (766/990) of facilities met these criteria, and facilities in both provinces requested additional training for an average of 3.7 staff members. This may be a result of varying skill levels and high turnover rates of end users. However, when comparing the knowledge and skills of a subset of NIIS end users, there was a clear correlation between the two. Hence, more and frequent refresher training is needed to ensure that end users can apply the knowledge and skills gained from training to their daily work. Findings from a recent study indicate that more time should be allocated for initial training, and training should be offered at various times to account for busy and demanding work schedules [2]. Trainings should also be revised over time, varied depending on the end users [2], and easy to replicate and roll out for end users across locations [15]. In addition, training using real-time data is often an overlooked factor that can empower users to track their own performance and, in turn, motivate peers [15]. In addition, facilities in remote areas or with limited resources can access remote e-learning via mobile apps or web-based platforms that have been shown to be less expensive but just as effective as traditional in-person training [16]. Furthermore, facilities can consider recognizing and rewarding high-performing staff in a transparent manner as a means to support, motivate, encourage, and hopefully retain end users [15,17,18].

With regard to system acceptance and feedback of end users for system improvement, 94.5% (725/767) of end users interviewed in both provinces reported that the NIIS supported them in managing and reporting immunization data and agreed that the quality of immunization data was better. These results are consistent with findings from Zambia, where 94% (83/89) of end users reported that data accuracy was good or excellent, and 28% (25/89) and 27% (24/89) reported an increase in their ability to identify areas with low vaccine coverage and children who have missed vaccines, respectively [3]. On the other hand, user satisfaction with an electronic medical record system use in 5 low-resource setting hospitals in Ethiopia was low with an overwhelming preference for paper-based records; respondents strongly disagreed that the system helped finish tasks faster or had a positive effect on the quality of care provided [13].

The finding that 4.3% (33/767) of end users reported that NIIS increased their workload is not surprising, as end users were purposefully asked to maintain paper-based records in addition to the NIIS primarily to identify bugs in the NIIS and update the system based on user feedback. A recent study on the analysis of EIR data in Tanzania showed that facilities that had transitioned to paperless reporting were more likely to use the EIR compared with facilities that were still responsible for reporting through parallel paper-based and paperless systems, when controlling for other factors. The authors hypothesized that this was mainly because of health care worker bandwidth and motivation [19]. Although the MOH had designated that on June 1, 2018, Vietnam would fully transition to only using the NIIS, there was no guidance on how this should be done. According to lessons learned from the Better Immunization Data initiative, overall data quality can suffer as a result of not setting expectations for both systems [20]. As expected, the mean time to create monthly immunization plans and reports after NIIS implementation on average was much lower than the mean time for these activities before NIIS implementation. These findings are consistent with results from Tanzania, where health care workers spent 41% less time registering and updating data, and as a result, saved 8 working days per year that could be reallocated to patient care [3]. Although the time spent on monthly planning and reporting was still higher than desired, this was mainly because of maintaining the NIIS along with the paper-based logbooks in parallel. However, as confidence in the NIIS increases and local authorities remove the paper-based system, end users will be able to fade out using the paper-based system, thus reducing the time spent on these activities.

Evaluation of the accuracy of data was encouraging, with 80.3% (795/990) of facilities reporting that the immunization data in the NIIS was accurate with the results from the paper system. This was validated upon further observation, where 88.6% (335/378) and 89.9% (242/269) of vaccination data matched between the NIIS and immunization cards and paper-based logbooks, respectively. In addition, vaccine stock counts were entered into the NIIS system at 90.8% (899/990) of the facilities. However, there is room for improvement; only 54.5% (206/378) of children had a matching ID on their immunization card and in the NIIS, 57% (13/23) of BCG vaccinations recorded in the NIIS were consistent with paper-based logbooks, and only 70% (21/30) of the facilities had consistent physical vaccine stock balances. These findings may be because of a delay in entering this information into the NIIS. Hence, the importance of the timeliness, completeness, and accuracy of the data should be reinforced regularly. Moreover, the feedback received from end users for system improvement suggests that there are still needs that should be addressed as soon as possible to ensure improvements in data quality and ultimately enable a transition to only using the NIIS.

It is important to highlight the importance of considering the feedback received from users on the need for more detailed guidelines and standard operating procedures across all facets of NIIS. User-based design has been the cornerstone of the successful expansion and use of digital health initiatives [15,21]. In Ghana, user-based design was critical in the expansion and rollout of telemedicine and led to the reduction of unnecessary hospital referrals [21].

Data storage, access, and confidentiality are additional essential components that can influence scale and transition [15]. End users can only use NIIS to access and enter the data into the system. The system was built to link tables and data elements or variables, and data dictionaries were aligned and formatted according to MOH national standards to allow for interoperability and the ability to connect with other systems. Per MOH regulations, built-in system user authentication allows users to tailor accessibility and permissions depending on their role and level. The NIIS database is hosted by Viettel, a state-owned company that hosts other government systems and is well known for its data security services. In addition, training includes components of user awareness and data protection and emphasizes that all users, regardless of role and level, are responsible for data accuracy, completeness, and privacy of data.

### Limitations

This study has several limitations. First, as only 2 provinces were selected, these findings may not be generalizable to the country as a whole. Second, the selection of facilities, children, and end users may not be representative of the overall population in Vietnam or other low to middle-income resource settings. Third, responses from the facility assessment, including infrastructure, capacity, and the need for human resources and NIIS end users’ perceptions and feedback, were self-reported by end users and, therefore, not objectively verified.

### Conclusions

This study showed that Ha Noi and Son La provinces in Vietnam were almost ready to fully transition to the NIIS in terms of NIIS use, infrastructure, and end users’ basic skills. However, there is still a need for additional support and resources to improve timeliness, completeness, and accuracy of the data and strengthen NIIS end users’ skills. In the meantime, it is imperative that any software issues with the NIIS are fixed, barcode IDs are issued for all children, and e-cards are launched soon so as to encourage NIIS uptake, especially in fee-based facilities, and to improve the data quality of the NIIS.

The design of the readiness assessment could be replicated in other provinces in Vietnam, as well as in other countries considering scale up of similar EIRs. Moreover, the findings from this study can inform other provinces in Vietnam or other countries on the types of challenges that must be addressed before fully transitioning to paperless reporting. As others have recommended [20], countries that are planning to scale an EIR should develop a clear roadmap for the eventual transition away from paper-based reporting, which may include plans for a readiness assessment.

## Abbreviations

BCG: Bacillus Calmette-Guérin
EIR: electronic immunization registry
EPI: expanded program on immunization
MOH: Ministry of Health
NIIS: National Immunization Information System
